# Ala54Thr Fatty Acid-Binding Protein 2 (*FABP2*) Polymorphism in Recurrent Depression: Associations with Fatty Acid Concentrations and Waist Circumference

**DOI:** 10.1371/journal.pone.0082980

**Published:** 2013-12-10

**Authors:** Roel J. T. Mocking, Anja Lok, Johanna Assies, Maarten W. J. Koeter, Ieke Visser, Henricus G. Ruhé, Claudi L. H. Bockting, Aart H. Schene

**Affiliations:** 1 Department of Psychiatry, Academic Medical Center, University of Amsterdam, Amsterdam, The Netherlands; 2 Program for Mood and Anxiety Disorders, University Center for Psychiatry UMCG, University of Groningen, Groningen, The Netherlands; 3 Department of Clinical Psychology, University of Groningen, Groningen, The Netherlands; CRCHUM-Montreal Diabetes Research Center, Canada

## Abstract

**Background:**

Fatty acid (FA)-alterations may mediate the mutual association between Major Depressive Disorder (MDD) and cardiovascular disease (CVD). However, etiology of observed FA-alterations in MDD and CVD remains largely unclear. An interesting candidate may be a mutation in the fatty acid–binding protein 2 (FABP2)-gene, because it regulates dietary FA-uptake. Therefore, we aimed to test the hypotheses that in MDD-patients the FABP2 Ala54Thr-polymorphism would be (I) more prevalent than in sex- and age-matched controls, (II) associated with observed alterations in FA-metabolism, and (III) associated with CVD-risk factor waist circumference.

**Methods:**

We measured concentrations of 29 different erythrocyte FAs, *FABP2*-genotype, and waist circumference in recurrent MDD-patients and matched never-depressed controls.

**Results:**

*FABP2*-genotype distribution did not significantly differ between the 137 MDD-patients and 73 matched controls. However, patients with the Ala54Thr-polymorphism had (I) higher concentrations of especially eicosadienoic acid (C20:2ω6; *P*=.009) and other 20-carbon FAs, and associated (II) lower waist circumference (*P*=.019). In addition, *FABP2-*genotype effects on waist circumference in patients seemed (I) mediated by its effect on C20:2ω6, and (II) different from controls.

**Conclusions:**

Although Ala54Thr-polymorphism distribution was not associated with recurrent MDD, our results indicate that *FABP2* may play a role in the explanation of observed FA-alterations in MDD. For Ala54Thr-polymorphism patients, potentially adaptive conversion of increased bioavailable dietary precursors into eicosadienoic acid instead of arachidonic acid might be related to a low waist circumference. Because this is the first investigation of these associations, replication is warranted, preferably by nutrigenetic studies applying lipidomics and detailed dietary assessment.

## Introduction

Major depressive disorder (MDD) - in particular its recurrent form (MDD-R) - is a major cause of disability and excess mortality worldwide [1]. The leading cause of this excess mortality in MDD is cardiovascular disease (CVD) [2]. Accordingly, the primary CVD-risk factor waist circumference - reflecting abdominal obesity and insulin resistance - is strongly associated with MDD [3–7]. Improved understanding of mechanisms underlying the MDD-CVD relationship might lead to novel life-prolonging (preventive) treatment strategies.

 An important mechanism underlying the MDD-CVD relationship may be fatty acid (FA)-metabolism, because it regulates e.g. inflammation, thrombosis and neurotransmitter-signaling [8–10]. FA-composition of cell membranes is altered in both MDD and CVD, with decreased ω3 polyunsaturated fatty acids (PUFAs) and increased ω6/ω3 PUFA ratios, e.g. increased arachidonic acid (C20:4ω6; ARA) relative to eicosapentaenoic acid (C20:5ω3; EPA) [8,11–13]. We corroborated these findings in a sample of 137 MDD-R patients, and extended them by showing additional changes in other FA-classes, e.g. lower overall FA-unsaturation, -chain length and -peroxidation [14,15]. However, etiology of FA-alterations in MDD and CVD remains largely unclear.

 FA-metabolism is influenced by many different factors, including genotype [16], dietary intake (e.g. essential ω3 FAs from fatty fish), lifestyle (physical activity, smoking), and hormonal regulation [14,17]. Interestingly, we previously reported that FA-alterations in MDD-R follow a bimodal distribution [18]. Instead of being unimodally distributed, with regard to FA-alterations, MDD-R-patients seemed to consist of two separate groups reflected by two unimodal distributions, a phenomenon also observed in schizophrenia [19]. This implies a dichotomous causal factor that divides patients in two groups and thereby underlies these bimodally distributed FA-alterations [19]. A possible example of such a dichotomous causal factor may be a mutation in a gene involved in FA-metabolism.

 An interesting location for such a mutation may be the fatty acid–binding protein 2 (*FABP2*) gene. *FABP2* is mainly expressed in small intestine enterocytes, where it is responsible for uptake of dietary FAs. A transition *G* to *A* at *FABP2*-codon 54 results in an alanine (Ala) to threonine (Thr) amino acid substitution (Ala54 to Thr54) [20]. This single nucleotide polymorphism is common, with a Thr54 allelic frequency of 30% in most populations, resulting in altered *FABP2* FA-affinity. Homozygous Thr54-carriers show altered dietary FA-uptake, with increased postprandial concentrations of 14–18-carbon fatty acids [21]. Because of the (patho)physiological role of FAs in metabolism, this altered FA-uptake has been suggested to explain the association of *FABP2* with increased insulin resistance and FA-oxidation, supporting observations suggesting a role of the *FABP2* Ala54Thr-polymorphism in CVD-etiology (e.g. increased waist circumference and atherosclerosis) [21–25].

 Considering this involvement of *FABP2* in FA-metabolism, the *FABP2* Ala54Thr-polymorphism may also be particularly interesting in the explanation of FA-metabolism alterations in MDD-R patients, because - as stated above - we observed (I) increased concentrations of 14–18-carbon FAs of several FA-subclasses [14], and (II) highly significantly lower overall FA-chain length [15], which was (III) bimodally distributed [18]. Surprisingly, previous studies in healthy and CVD-populations found no consistent associations of FA-concentrations with the *FABP2* Ala54Thr-polymorphism [24,26,27]. This may be because, to our knowledge, not all members of the different FA-subclasses (e.g. long chain saturated and monounsaturated FA’s) were studied, especially not in a specific psychiatric (e.g. depressed) population with known bimodally distributed FA-alterations.

 Therefore, the aim of the present study was to test the hypotheses that in MDD-R-patients the Thr54-polymorphism in the *FABP2*-gene would be (I) more prevalent than in sex-and age-matched controls, (II) associated with observed (bimodally distributed) alterations in FA-metabolism, and (III) associated with CVD-risk factor waist circumference.

## Materials and Methods

### 2.1. Ethics Statement

All participants provided written informed consent prior to enrolment. The ethics committee of the Academic Medical Center of the University of Amsterdam approved the study.

### 2.2. Subjects

The current study was part of the DELTA-study, a randomized clinical trial on the effect of an 8-week cognitive therapy on MDD-recurrence, described previously [28,29]. This trial has been registered in the ISRCTN registry as ISRCTN68246470. As part of DELTA-study’s 2-year follow-up measurements, we invited subjects to participate in the current study. At the start of the DELTA-study, participants had to (I) be aged between 18 and 65, (II) have had ≥2 previous major depressive episodes in the last five years, and (III) be in remission of MDD. Exclusion criteria were current or previous mania or hypomania, any psychotic disorder, alcohol or drug abuse, and predominant anxiety disorder. Since any form of therapy (e.g. antidepressant-use) was no inclusion or exclusion criterion for the trial, the DELTA-sample can be considered representative for MDD-R patients with respect to this characteristic.

 In addition to the MDD-R patient sample, we recruited age- and gender- matched healthy control subjects, without a personal and/or family history of MDD, as described previously [14].

### 2.3. Measurements

We took 20ml blood by venipuncture from subjects in the nonfasting state. As a model of brain FA-concentrations, we used washed erythrocytes, stored at −80 °C until analyses by capillary gas chromatography, as described previously [14]. This resulted in data on 29 different FAs, expressed in pmol/10^6^ erythrocytes [15]. We operationalized FA-metabolism in two steps. First we tested five main FAs: linoleic acid, arachidonic acid, α-linolenic acid, eicosapentaenoic acid and docosahexaenoic acid, together with three indices which describe overall FA-characteristics: the Unsaturation (UI), Peroxidation (PI) and Chain Length Indices (CLI) [15,30]. Subsequently, we exploratively tested 24 other FAs. This approach reduces the multiple testing problem, because it guides interpretation of effects as explorative or *a priori* selected [15,31].

 DNA was isolated from blood using a filter-based method (QIAamp DNA Mini Kit, Qiagen Ltd, United Kingdom). Polymerase chain reaction (PCR) primers were designed using Primer 3. PCR primer sequence TGACAATTTGAAGCTGACAATTA and AATCAAGAATGCATTGCTCAT, PEX primer sequence bioAT AAA TTC ACA GTC (L)AA GAA TCA AGC. Genotyping was done using a Matrix Assisted Laser Desorption Ionization Time Of Flight (MALDI-TOF) mass spectrometer from Bruker Daltonics [32]. All samples were genotyped in duplicate to increase reliability. Genotyping error rate based on these duplicates was 3.7%. We operationalized *FABP2*-genotype in three categories: *GG* homozygous, *AG* heterozygous and *AA* homozygous. An *A*-allele results in the Thr54-polymorphism.

 We determined waist circumference in centimeters using a standard operating procedure [3,4].

### 2.4. Statistical analyses

#### 2.4.1. Missing data

We used multiple imputation to prevent bias possibly introduced by missing or non-detectable FA- or genotype-data, which resulted in 5 imputed datasets as described previously [15,33].

#### 2.4.2. Hardy-Weinberg Equilibrium (HWE)

We tested deviations from HWE separately in patients and controls using an online calculator χ^2^-test, available on http://www.oege.org/software/hwe-mr-calc.html. 

#### 2.4.3. Subject characteristics

We compared patients’ and controls’ subject characteristics using χ^2^-tests or independent Student’s t-tests as appropriate.

#### 2.4.4. Hypotheses testing

For the first hypothesis, we used χ^2^-tests to test whether the distribution of observed genotype frequencies for patients and controls differed from expected frequencies. In order to test the association of the Thr54-polymorphism with the bimodally distributed FA-alterations in MDD-R-patients (2^nd^ hypothesis), we used linear mixed models with genotype as predictor variable and a FA-concentration as outcome variable [34]. In case the overall F-test for a given FA was significant, we reported parameter estimates for the distinct genotypes with *GG* as reference category. For the association of Thr54-polymorphism with CVD-risk (3^rd^ hypothesis), we applied a similar model, except that we entered waist circumference as outcome variable. We performed no correction for confounders, because these effects concern genetic effects and genotype is not expected to be subject to confounding factors.

 In addition, we planned several post-hoc tests. First, in case FA-alterations would show linear associations with *FABP2* genotype, e.g. GG: lowest value, AG: middle value, AA, highest value, we would test the effect of *FABP2* on FA-alterations in linear mixed models with *FABP2* recoded as scale variable as predictor variable and the FAs as outcome variable. Second, if FA-concentrations and waist circumference would be associated with *FABP2*-genotype, we would test correlations between these FA-alterations and waist circumference. If these alterations would be significantly correlated, in order to distinguish the direction of these effects, we would perform mediation analyses. To this end, we would use linear mixed models, first with *FABP2* and waist circumference as predictor variables and the FA as outcome variable, subsequently with *FABP2* and the FA as predictor variables and waist circumference as outcome variable. If the effect of *FABP2* on the FA or waist circumference would disappear after inclusion of the other factor (the FA or waist circumference) in the model, this would imply that this other factor mediates the influence of *FABP2*. Finally, third, if *FABP2* would affect FA-concentrations or waist circumference in the MDD-patients, we would test whether this effect would differ from the effect in the matched controls. In order to test this, we would build another set of linear mixed models with *FABP2* and patients-status (patient or control) and their interaction (*FABP2*×patient-status) as predictor variables and the FA or waist circumference as outcome variable. Because the patient-status factor in these models may be subject to confounding, in contrast to the genetic *FABP2*-factor, we corrected observed effects for possibly confounding differences between patients and controls in age, sex, marital status, educational level, social class, ethnicity, 17-item Hamilton depression rating scale (HDRS)-score, and smoking. To prevent losing power, we used propensity scores, which enable correction for multiple potentially confounding factors while retaining power. Propensity scores represent for each case the saved predicted probability of being a patient or a control, which we calculated using a binary logistic model with patient-status as dependent, and the chosen potential confounders as predictor variables.

 Although often not dealt with in FA-research, the multitude of FAs makes multiple comparisons inherent in investigating FA-metabolism, potentially causing type-I errors [15]. To reduce this problem we (I) applied pathophysiologically driven data reduction using the UI, CLI and PI (10), and (II) a priori designated several outcomes as hypothesis based, and others as explorative. Although still subject to debate, Bender and Lange (2001) suggest to perform correction for multiple testing primarily in confirmatory studies, while explorative results should be clearly indicated as such [31]. In line with their advice, and because e.g. Bonferroni correction likely would be too strict considering the relatively strong assumed correlations between the different outcomes in this study and therefore may induce type-II errors, we consequently chose to correct neither the results of the a priori hypothesized outcomes, nor the explorative results, for multiple comparisons [31]. Therefore, particularly the explorative results of this first investigation of associations between *FABP2* and several FAs - particularly in a psychiatric population - should be interpreted as such.

#### 2.4.5. Power analyses

We performed sensitivity power analyses. With power=.80 and two-sided alpha=.05, we were able to detect effects with a small effect size (w>0.214;f>0.216;f^2^>0.080) for all analyses, except for the differences between the different genotype classes in FA-measures and waist circumference in the patient group, for which we were able to detect medium effect sizes (f>0.268).

#### 2.4.6. Software

We performed multiple imputation using the package Amelia II, implemented in R [33]. We performed analyses using SPSS Statistics v.20 (IBM). For multiple imputation results that are not pooled by SPSS, we used Rubin’s rules [35]. For the linear mixed models, these were implemented in a macro available at http://www.amcpsychiatrie-depressie.nl/cms/downloadfile.asp, as described previously. We used G*Power 3.1.3 (Kiel, Germany) to perform power calculations.

## Results

### 3.1. Included sample and missing data

The inclusion procedure resulted in 137 patient and 73 control participants. Of these subjects, 8 patients and 3 controls had no valid *FABP2-*genotype due to technical reasons. Missingness in FA-data has been described previously [15]. Standard diagnostics by Amelia II suggested successful imputation.

### 3.2. Subject characteristics

*FABP2*-genotype was in HWE in controls (*P*>.05). Equal sex and age distributions among patients and controls indicate successful matching (*P*>.05). Patients had lower educational level (*P*<.001) and greater waist circumference (*P*=.025) (Table S1).

### 3.3. FABP2-distribution in patients and controls

The distribution of *FABP2*-genotype did not differ among patients and controls (*P*=.627). In patients, genotype frequencies were *GG*: 57.7%, *AG*: 34.3%, and *AA*: 8.0%, and in controls 50.7%, 41.6%, and 7.7%, respectively.

### 3.4. Association FABP2-FA-concentrations

**Figure 1:**
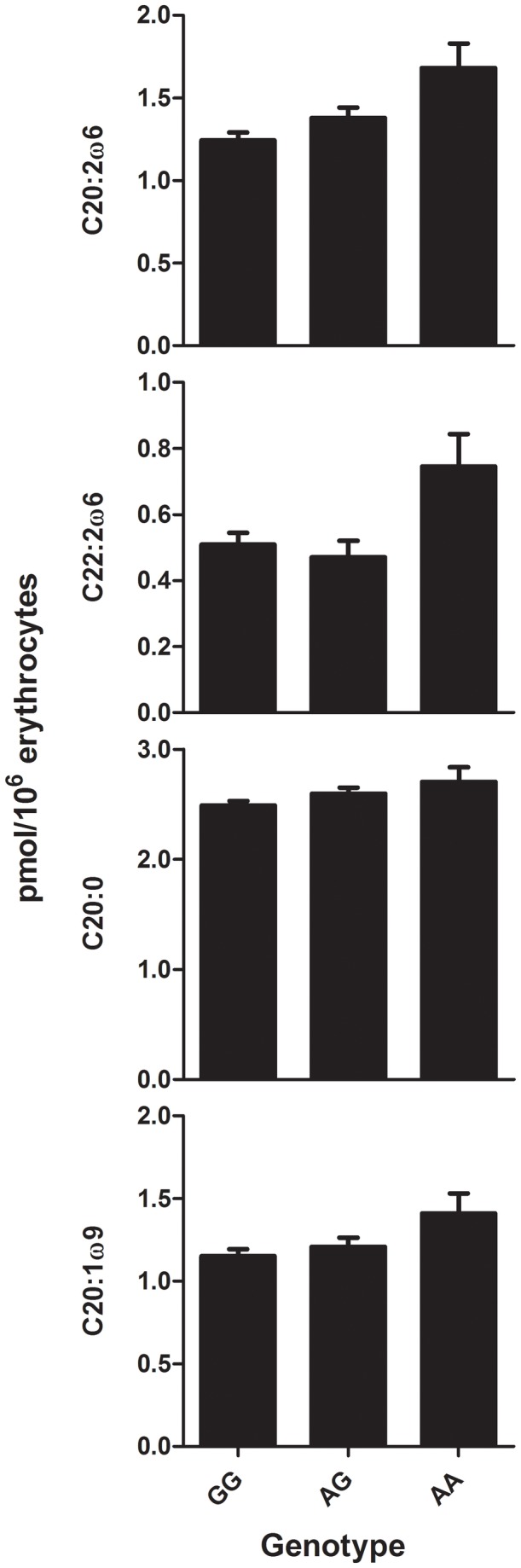
Fatty acid concentrations in recurrent depression according to FABP2 genotype. Concentrations of four fatty acids [eicosadienoic acid (C20:2ω6), docosadienoic acid (C22:2ω6), arachidic acid (C20:0) and gondoic acid (C20:1ω9)] in 137 recurrently depressed patients according to Ala54Thr fatty acid-binding protein 2 (FABP2) polymorphism genotype (GG, AG, AA).

Regarding the association of the Thr54-polymorphism with FA-metabolism, MDD-R patients’ FA-concentrations of the *a priori* selected five main FAs and three indices did not differ according to *FABP2*-genotype (Table 1). In the explorative analyses of the other FAs, two FA-concentrations were significantly associated with *FABP2-*genotype: eicosadienoic acid (C20:2ω6; F_2,222.6_=4.801,*P*=.009) and docosadienoic acid (C22:2ω6; F_2,289.1_=3.271,*P*=.039; Table 1). Looking at the parameter estimates for the three subgroups, the *AA*-genotype had higher eicosadienoic acid (*b*=0.437,*SE*_*b*_=0.156,*t*=7.886,*P*=.006) and docosadienoic acid-concentrations (*b*=0.236,*SE*_*b*_=0.120,*t*=2.324,*P*=.020) compared to the reference *GG*-category (Figure 1). 

**Table 1:** Differences in fatty acid concentrations, fatty acid indices and waist circumference between *GG*, *AG* and *AA*-carriers of the Ala54Thr fatty acid-binding protein 2 (*FABP2*) polymorphism, vertically graphically divided in *a* *priori* (upper panel) and explorative (lower panel) tests.

	***GG (N=79)^a^***	***AG (N=47)^a^***	***AA (N=11)^a^***	***F*-value**	***Df1***	***Df2***	***P*-value**
**C18:2n6**	65.42±1.528	67.05±1.922	65.64±4.198	0.215	2	87.502	.807
**C20:4n6**	71.07±0.976	73.51±1.374	71.78±2.819	1.003	2	44.068	.375
**C18:3n3**	0.826±0.036	0.845±0.044	0.848±0.091	0.073	2	163.23	.930
**C20:5n3**	3.381±0.188	3.278±0.270	3.381±0.534	0.049	2	56.873	.952
**C22:6n3**	15.19±0.558	14.63±0.844	14.16±1.535	0.287	2	38.681	.752
**PI**	1.101±0.012	1.096±0.016	1.107±0.031	0.057	2	106.47	.945
**CLI**	18.31±0.023	18.30±0.030	18.38±0.065	0.601	2	164.04	.549
**UI**	1.290±0.009	1.289±0.012	1.305±0.023	0.187	2	125.27	.830
**WC**	91.40±1.556	88.04±2.010	79.16±4.173	4.000	2	896.61	**.019**
**C14**	3.333±0.116	3.287±0.154	2.817±0.351	1.047	2	83.660	.355
**C16**	162.8±2.597	165.5±3.300	157.9±7.915	0.466	2	90.552	.629
**C18**	103.0±1.206	104.6±1.554	100.4±3.338	0.761	2	295.71	.468
**C20**	2.490±0.042	2.597±0.054	2.705±0.132	1.965	2	46.139	.152
**C22**	7.568±0.190	7.481±0.234	8.287±0.501	1.085	2	120.20	.341
**C24**	14.78±0.496	14.64±0.653	16.40±1.378	0.727	2	84.733	.486
**C18:4n3**	0.221±0.028	0.280±0.037	0.246±0.078	0.777	2	133.36	.462
**C22:5n3**	8.009±0.196	7.913±0.251	7.918±0.543	0.048	2	87.828	.953
**C18:3n6**	0.583±0.026	0.589±0.030	0.479±0.083	0.906	2	24.758	.417
**C20:3n6**	8.879±0.284	8.796±0.376	9.738±0.887	0.536	2	40.268	.589
**C22:4n6**	10.46±0.310	10.67±0.409	11.64±0.848	0.883	2	153.36	.416
**C22:5n6**	1.695±0.070	1.738±0.095	1.777±0.179	0.126	2	62.253	.882
**C20:2n6**	1.243±0.049	1.377±0.065	1.680±0.148	4.801	2	222.65	**.009**
**C22:2n6**	0.509±0.036	0.470±0.050	0.745±0.098	3.271	2	289.13	**.039**
**C14:1n5**	0.288±0.036	0.308±0.044	0.286±0.110	0.055	2	40.243	.947
**C16:1n7**	3.181±0.172	3.068±0.262	2.986±0.521	0.099	2	40.381	.906
**C18:1n7**	7.469±0.169	7.651±0.246	7.162±0.533	0.403	2	38.358	.671
**C20:1n7**	0.254±0.029	0.304±0.038	0.261±0.100	0.443	2	41.976	.645
**C16:1n9**	0.798±0.149	1.124±0.204	1.078±0.543	0.730	2	13.743	.500
**C18:1n9**	74.61±1.183	74.92±1.563	73.49±3.390	0.075	2	98.188	.927
**C20:1n9**	1.151±0.042	1.207±0.056	1.409±0.123	2.099	2	62.402	.131
**C22:1n9**	1.792±0.241	2.042±0.317	2.010±0.652	0.219	2	1928.1	.803
**C24:1n9**	13.16±0.415	13.25±0.541	14.52±1.208	0.624	2	87.715	.538
**C20:3n9**	0.397±0.028	0.371±0.036	0.243±0.076	1.849	2	101.56	.163
**Total FAs**	585.5±6.571	593.3±8.685	573.3±19.72	0.527	2	984.82	.590

Abbreviations: PI, Peroxidation Index; CLI, Chain Length Index; UI Unsaturation Index; WC, Waist circumference

^a^ N per genotype based on estimation after multiple imputation.

### 3.5. Association *FABP2*-waist circumference

**Figure 2:**
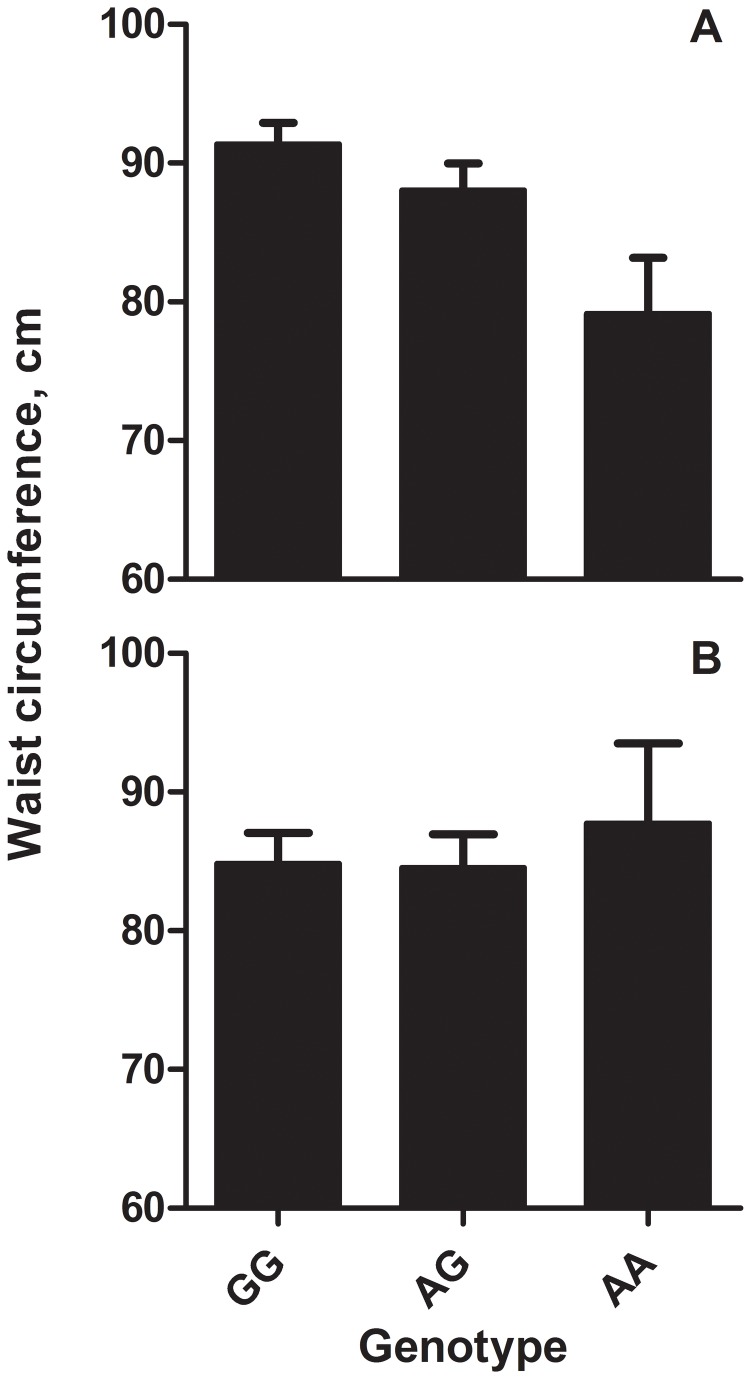
Waist circumference according to FABP2 genotype compared between recurrently depressed patients and healthy controls. Waist circumference according to Ala54Thr fatty acid-binding protein 2 (FABP2) polymorphism genotype (GG, AG, AA) in 137 recurrently depressed patients (Panel A) and 73 non-depressed controls (Panel B).

Considering the third hypothesis, MDD-R patients’ waist circumference significantly differed according to *FABP2*-genotype (*F*_2,896,_6=4.000,*P*=.019). *AA*-genotype was associated with lower waist circumference estimate compared to the reference GG category (*b*=-12.243,*SE*_*b*_=4.474,*t*=-2.737,*P*=.006; Figure 2, Panel A).

### 3.6. Post-hoc analyses

#### 3.6.1. Tests for linear FABP2–FA-associations

In the analyses of the association of the Thr54-polymorphism with FA-metabolism, we observed that while overall effect of genotype was not significant, parameter estimates for specific genotypes on some FAs were significant (*P*<.05) and suggested a linear associations of FA-concentrations across *FABP2* genotype, e.g. *GG*: lowest value, *AG*: middle value, *AA*, highest value. This affected especially FAs with a 20-carbon chain length. We therefore tested this linear relationship using linear mixed models with *FABP2*-genotype recoded as a scale variable (*GG*=1, *AG*=2, *AA*=3). These models showed significant linear effects of *FABP2*-genotype on C20 (*b*=0.106,*SE*_*b*_=0.052,*t*_135_=2.032,*P*=.045), C20:2ω6 (*b*=0.182,*SE*_*b*_=0.062,*t*_135_=2.944,*P*=.004), and C20:1ω9 (*b*=0.099,*SE*_*b*_=0.049,*t*_135_=2.001,*P*=.046; Figure 1).

#### 3.6.2. Relationship FABP2, FA-concentrations, and waist circumference

In order to disentangle *FABP2*’s effects on FA-concentrations and waist circumference, we performed correlation-analyses between waist circumference and FA-concentrations, and mediation analyses concerning the effect of *FABP2* on FA-concentrations and waist circumference. In line with the effects of *FABP2*-genotype on both eicosadienoic acid and waist circumference, a post-hoc Pearson’s correlation test showed that eicosadienoic acid (*r*=-.243, *P*=.005) was significantly negatively correlated with waist circumference in patients. Subsequently, in a linear mixed model with both *FABP2*-genotype and waist circumference as predictors of eicosadienoic acid, we observed that both *FABP2* (F_2,317.47_=3.285,*P*=.039,*b*_AA vs. GG_=0.362,*SE*_*b*_=0.155,*t*_173.915_=2.342,*P*=.020) and waist circumference (F_1,3184.09_=4.790,*P*=.029,*b*=-0.006,*SE*_*b*_=0.003,*t*_3184.09_=-2.189,*P*=.029) independently predicted eicosadienoic acid concentrations (overall F_3,799.79_=4.890,*P*=.002). However, the other way around, the predictive effect of *FABP2* on waist circumference lost significance (*P*=.102) after including eicosadienoic acid as a [significant (*P*=.031)] predictor in the model. This indicates that *FABP2*’s effect on waist circumference is mediated by its effect on eicosadianoic acid, i.e. *FABP2*’s effect on waist circumference seems to consist of higher eicosadienoic acid concentrations in A-allele carriers which on their turn result in lower waist circumference.

#### 3.6.3. Interactions between patient-status and FABP2 on C20:2ω6 and waist circumference

To test whether the relation between *FABP2* and C20:2ω6 and waist circumference differed between patients and controls, we performed linear mixed models with patient-status (yes/no) and *FABP2*-genotype (GG/AG/AA) and their interaction as predictor variables, and C20:2ω6 or waist circumference as outcome variables. There was no patient-status×*FABP2*-genotype interaction for C20:2ω6 (*F*_2,882,_788=1.069, *P*=.344). For waist circumference, the interaction was also non-significant (*F*_2,640,047_=2.055, *P*=.129), but the patient×AA-genotype parameter estimate was significant at trend level (*b*=-15.140,*SE*_*b*_=7.719,*t*_3184.09_=-1.961,*P*=.051), suggesting that in patients *AA*-genotype was associated with a relatively lower waist circumference compared to controls (Figure 2, Panel A and B). After correction - using propensity scores - for possibly confounding differences between patients and controls in e.g. educational level, social class, HDRS-score, and smoking, the overall genotype effect remained non-significant (*F*_2,477,_338=2.129, *P*=.120). However, the patient×AA-genotype parameter estimate now just gained significance (*b*=-15.681,*SE*_*b*_=7.946,*t*_242.59_=-1.974,*P*=.050).

## Discussion

### 4.1. Summary of results

In the present paper, we examined *FABP2*’s role in MDD-R, and particularly its relation with bimodally distributed FA-alterations and CVD-risk factor waist circumference. *FABP2* Ala54Thr-polymorphism distribution did not differ between 137 patients with recurrent MDD and 73 matched healthy controls without an MDD-history. However, *A*-allele carrying MDD-R patients had (I) higher concentrations of several 20-carbon FAs, especially eicosadienoic acid (C20:2ω6), and associated (II) lower waist circumference. In addition, post-hoc analyses suggested that effects of *FABP2* on waist circumference in patients (I) were mediated by its effect on C20:2ω6, and (II) were different from controls.

### 4.2. FABP2 genotype distribution

Although it is increasingly recognized that FA-metabolism may play an important role in psychiatric disease, this is – to the best of our knowledge – the first investigation of the role of *FABP2* in a psychiatric population. Contrary to our first hypothesis, genotype distribution did not differ from matched controls. This may imply that the Ala54Thr-polymorphism plays no role in vulnerability for MDD-R, as opposed to what have been suggested for other disorders including obesity and atherosclerosis [21–25].

### 4.3. Association between FABP2 and FA-metabolism

However, with regard to our second hypothesis, although the earlier observed bimodally distributed FA-metabolism parameters [18] were not associated with the Ala54Thr-polymorphism, we observed specific associations of the *A*-allele with the biologically mutually related FAs eicosadienoic acid (C20:2ω6) and its elongation product docosadienoic acid (C22:2ω6) [14]. In addition, also other 20-carbon FAs (C20, C20:2ω6, and C20:1ω9) showed a significant linear relation of increased concentrations with one or two *A*-alleles. Interestingly, previous research in healthy and CVD-subjects did not find consistent associations of the Ala54Thr-polymorphism with FA-concentrations [24,26,27]. This could be explained by lack of power [24,27], use of other sample mediums [24,26,27], lack of measurement of eicosadienoic acid (C20:2ω6) and docosadienoic acid (C22:2ω6), or inclusion of different patient populations [24,26,27].

 Regarding other FAs, two of these previous studies found no associations [24,26], while one other study [27] found lower palmitoleic acid (C16:1ω7) and higher α-linolenic (C18:3ω3) and lignoceric acid (C24:0) in phospholipids to be associated with threonine coding *FABP2*-alleles. Interestingly, a study investigating *FABP2*’s effect on FA-uptake suggested that threonine coding *FABP2*-alleles are associated with an increased uptake of particularly 14-18-cabon FAs, including α-linoleic acid (C18:2ω6) [21], which can be enzymatically elongated to eicosadienoic acid (C20:2ω6) and docosadienoic acid (C22:2ω6) [14]. It may therefore be hypothesized that the increased red blood cell membrane concentration of eicosadienoic acid in our patients with the *AA*-genotype may reflect an adaptive conversion of increasedly bioavailable α-linoleic acid (C18:2ω6), to prevent accumulation of its other, and supposedly more bioactive (as main precursor of pro-inflammatory eicosanoids), conversion product arachidonic acid (ARA; C20:4ω6) [14]. Of note in this respect, increased ARA has been associated with (visceral) obesity and CVD-risk [36,37].

 Interpretation of increased eicosadienoic acid concentrations in *AA*-genotype patients as an adaptive process to prevent ARA-accumulation corresponds with the observed negative association of eicosadienoic acid and waist circumference. In addition, eicosadienoic acid mediated the lower waist circumference in *AA*-patients: the relation between *FABP2* and waist circumference lost significance when eicosadienoic acid was incorporated in the model. This might imply that for *AA*-patients, conversion of increasedly bioavailable α-linoleic acid (C18:2ω6) into eicosadienoic acid (C20:2ω6) instead of ARA (C20:4ω6) helps in maintaining a low waist circumference thereby possibly reducing CVD-risk.

### 4.4. Association between FABP2 and waist circumference

Our finding of an interaction at trend level between patient-status and *FABP2*-genotype on waist circumference is intriguing. Contrary to our third hypothesis and results in controls, *AA*-genotype was significantly associated with a lower waist circumference in the MDD-patients. This may have several explanations. First, patients’ metabolic constitution may be influenced by other genes, e.g. mutations in the 1-carbon-cycle, interacting with *FABP2*’s effect on waist circumference [38]. In addition, stress may affect FA-metabolism leading to different associations in patients, through the association between the HPA-axis and FA-metabolism [39]. Finally, *FABP2*’s effects may interact with dietary availability of nutrients, which could differ between patient and controls [16]. Future research should investigate these possibilities.

 Interestingly, several studies also indicate that the Ala54Thr-polymorphism may be associated with a beneficial CVD-risk profile in response to certain dietary regimens, including eicosapentaenoic acid supplementation [40], or moderate-fat or high-polyunsaturated fat diets [41,42]. In these studies, Ala54Thr-polymorphism carriers had better metabolic responses and e.g. a larger reduction in waist circumference. This may indicate that different dietary preferences in patients played a role in the interaction between patient-status and *FABP2* on waist circumference. Unfortunately, a main limitation of the present study is that no dietary data has been collected. Nevertheless, given the genetic nature of our observed effects, dietary intake likely is not a confounding factor, but rather interacts with *FABP2*-genotype to explain observed relations. In addition, erythrocyte FA-concentrations are thought to be more stable than plasma FAs, thereby reflecting long-term FA-metabolism, instead of dietary fluctuations [17]. Future nutrigenetic studies combining *FABP2* and dietary assessment will further elucidate their interaction in explaining altered FA-metabolism and waist circumference in MDD(-R).

### 4.5. Additional limitations

Some additional limitation should be mentioned. Analyses did not differentiate between FA-subclasses, e.g. sphingomyelin or phosphatidylcholine. More advanced lipidomic analyses may provide better insight into the impact of *FAB2* on FA-metabolism. However, this is the first report including a wide range of different FAs and indices into the analyses. This brings up another important point: the issue of multiple testing. Because of the multitude of FAs, studying FA-metabolism usually entails multiple comparisons. Although often overlooked in FA-literature, multiple comparisons may lead to type-I errors [15]. We dealt with this intrinsic problem by (I) applying pathophysiologically driven data reduction using the UI, CLI and PI [15], and (II) *a priori* designating several outcomes as hypothesis based, and others as explorative. However, despite these precautions, it remains important to keep the multiple testing problem in mind when interpreting results. None of the reported significant results would have survived Bonferroni correction. However, this correction likely would have been too strict considering the relatively strong assumed correlations between the different outcomes in this study. For that reason, strict correction in this sample could have induced type-II errors, and therefore we consequently chose to correct neither the results of the *a priori* hypothesized outcomes, nor the explorative results, for multiple comparisons [31]. Therefore, particularly explorative first results should be interpreted as such, and replication in further investigations is warranted. In addition, while power calculations showed that we had adequate power to detect small effect sizes for almost all analyses, we had insufficient power to detect small effect sizes in the analyses on the differences between the different genotype classes in FA-measures and waist circumference in the patient group, for which we were able to detect medium effect sizes (f>0.268). Therefore, we may have missed additional small effects of *FABP2* on FA-measures or waist circumference. However, for these analyses, we additionally operationalized the effects of *FABP2* as a linear function, which resulted in adequate power to detect small effect sizes (f^2^>0.080). So, in conclusion, the only effects for which our set-up may have precluded detection of small effect sizes would be non-linear effects of *FABP2* on FA-measures and/or waist circumference. However, both (I) existing literature on the physiological role of *FABP2*, and (II) data from our sample presented in table 1, do not suggest such non-linear relationships.

### 4.6. Study strengths

Our study also has particular strengths. To our knowledge *FABP2* was investigated in a psychiatric population for the first time, in a specific sample of MDD-R-patients with known bimodally distributed FA-alterations. In addition, an advanced multiple imputation procedure reduced the possibility that missing data have influenced results [15,33]. Finally, this is the first report of an association between *FABP2* and two specific mutually biologically related FAs, namely eicosadienoic acid (C20:2ω6) and docosadienoic acid (C22:2ω6), which increases knowledge on *FABP2*’s role in health and disease.

## Conclusion

Although Ala54Thr-polymorphism distribution was not associated with MDD, *A*-allele carrying patients had (I) higher concentrations of several 20-carbon FAs, particularly eicosadienoic acid (C20:2ω6), and associated (II) lower waist circumference. Therefore, for *AA*-patients, potentially adaptive conversion of increasedly bioavailable dietary precursors into eicosadienoic acid might mediate maintenance of a low waist circumference, which may guide future investigations of CVD-prevention in these patients. Considering the explorative nature of this first investigation of these associations, replication is warranted, preferably by nutrigenetic studies applying lipidomics and detailed dietary assessment.

## Supporting Information

Table S1 **Subject Characteristics.** ^a^ Educational level is defined as: low, primary education or preparatory middle-level applied education; middle, higher general continued education or middle-level applied education; and high, preparatory scientific education, higher applied education, or scientific education. ^b^ based on occupation: Class 1, e.g. cleaner; Class 2, e.g. nurse; Class 3, e.g. general manager. Abbreviations: HDRS, Hamilton depression rating scale; TCA, tricyclic antidepressant; SSRI, selective serotonin reuptake inhibitor.
(DOCX)
