# Supplementary material for: Ala54Thr Fatty Acid-Binding Protein 2 (FABP2) Polymorphism in Recurrent Depression: Associations with Fatty Acid Concentrations and Waist Circumference
Source: PLoS One. 2013 Dec 10;8(12):e82980. doi: 10.1371/journal.pone.0082980 (PMC3858331; doi:10.1371/journal.pone.0082980)
Supplement: Table S1 — Subject Characteristics. a Educational level is defined as: low, primary education or preparatory middle-level applied education; middle, higher general continued education or middle-level applied education; and high, preparatory scientific education, higher applied education, or scientific education. b based on occupation: Class 1, e.g. cleaner; Class 2, e.g. nurse; Class 3, e.g. general manager. Abbreviations: HDRS, Hamilton depression rating scale; TCA, tricyclic antidepressant; SSRI, selective serotonin reuptake inhibitor. (DOCX) [file pone.0082980.s001.docx]

**Table S1.** Subject Characteristics

| **Characteristic** | **Patients  (*n* = 137)** | **Controls  (*n* = 73)** | ***p* Value** |
| --- | --- | --- | --- |
| Female, % | 73.7 | 69.9 | .55 |
| Age, mean (SE), year | 46.4 (0.8) | 44.7 (1.1) | .205 |
| Educational level^a^, % |  |  | <.001 |
| Low | 33.3 | 5.2 |  |
| Middle | 31.2 | 22.5 |  |
| High | 35.5 | 72.3 |  |
| Social class^b^, % |  |  | <.001 |
| Class 1 | 11.1 | 55.0 |  |
| Class 2 | 52.1 | 32.0 |  |
| Class 3 | 36.7 | 13.0 |  |
| Smoking, % | 49.0 | 39.4 | .21 |
| Waist circumference, mean (SE), cm | 89.3 (1.19) | 84.9 (1.42) | .025 |
| HDRS_17_ score, mean (SE) | 5.9 (.46) | 1.2 (.48) | <.001 |
| Current depressive episode, % (n) | 19.0 (26) | NA |  |
| Antidepressant use, % (n) | 62.8 (86) | NA |  |
| Number of previous episodes, mean (SE) | 7.71 (0.76) | NA |  |
| Age of first onset, mean (SE), year | 28.4 (1.08) | NA |  |
| TCA, % (n) | 5.1 (7) | NA |  |
| SSRI, % (n) | 38.7 (53) | NA |  |
| Other, % (n) | 19.0 (26) | NA |  |
